# Metronomic regimen as an effective treatment for aggressive T-LGL leukemia with central nervous system infiltration: clinical experience and review of literature

**DOI:** 10.18632/oncotarget.15762

**Published:** 2017-02-27

**Authors:** Yun Liu, Lei Fan, Huihui Zhao, Wei Xu, Jianyong Li

**Affiliations:** ^1^ Department of Hematology, The First Affiliated Hospital of Nanjing Medical University, Jiangsu Province Hospital, Nanjing, China; ^2^ Department of Hematology, The Second Hospital of Nanjing, Nanjing, China

**Keywords:** T-LGL leukemia, HLH, metronomic regimen

## Abstract

A 71-year-old man was diagnosed with T-Large granular lymphocytic (LGL) leukemia, which usually represents a relatively indolent clinical course. While the clinical manifestation of this patient we report herein was aggressive with lasting fever, splenomegaly and hemophagocytic lymphohistiocytosis (HLH). T-cell immunophenotype was CD3+CD4-CD8-CD5-CD7-TCRαβ+. After comprehensive evaluation, an adjusted chemotherapy regimen CEOP (cyclophosphamide, vincristine, etoposide, prednisone) with etoposide, a potential effective regimen for HLH was administrated to the patient. Although he received intensive regimen, the patient showed drug resistance and disease progression with central nervous system (CNS) involvement during treatment and showed only transiently response to intrathecal methotrexate, cytarabine and dexamethasone. Therefore, considering the refractory elderly patient with fragile physical condition, metronomic regimen T-PEPC (oral administration of thalidomide, prednisone, cyclophosphamide, etoposide and methylhydrazine) was recommended, which refers to the frequent even daily administration of cytotoxic drugs at comparatively low doses with minimal or prolonged drug-free breaks. The patient responded well to this treatment and remained symptom-free for 8-month follow-up. To our knowledge, this is the first case of reporting this unique immunophenotype of dual CD4-/CD8- with aggressive clinical course and CNS involvement that successfully treated with metronomic regimen, suggesting that low dose metronomic regimen could be a better option for elderly patient with aggressive T-LGL leukemia.

## INTRODUCTION

Large granular lymphocytic(LGL) leukemia is a rare T or NK clonal lymphoproliferative disorder, whose clinical presentation is dominated by a variety of autoimmune disorders including pure red cell aplasia, Sjogrens Syndrome and rheumatoid arthritis (RA) [1]. According to the world health organization (WHO) classification, LGL leukemia can be divided into CD3 (+) T-cell LGL (T-LGL) leukemia and CD3 (-) natural killer LGL (NK-LGL) leukemia subtypes [2]. The typical phenotype of T-LGL leukemia is CD3+/TCR-αβ+/CD4-/CD8+/CD57+/ CD16+ [3]. Usually, T-LGL leukemia is a clinically indolent disease and it represents the most common LGL disorder in western countries, according to 85% of all cases [4, 5]. Herein, we describe a unique case of 71-year-old male patient with aggressive T-LGL leukemia, who failed to respond and progressed with CNS involvement during induction chemotherapy and achieved durable disease control with metronomic regimen.

## CASE REPORT

**Figure 1:**
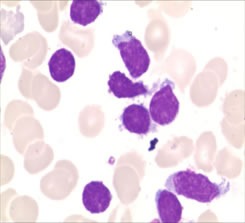
The neoplastic lymphoid cells in bone marrow aspirates with irregularly shaped nuclei, a moderate amount of cytoplasm, and large cytoplasmic granules (Wright-Giemsa stain, ×1,000).

**Figure 2:**
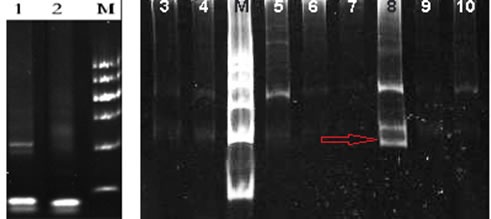
TCR gene rearrangement test in our hospital: TCR gene rearrangement by PCR was positive for TCR β and γ (red arrow, lane 8)

**Figure 3:**
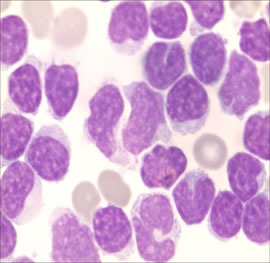
Cerebrospinal fluid test: massive amount of cytoplasm and large cytoplasmic granules, with irregular shaped nuclei in cerebrospinal fluid (Wright-Giemsa stain, ×1, 000).

A 71-year-old man who presented with persistent left-sided abdominal pain and hyperpyrexia for two months was admitted into local hospital. Complete blood cell (CBC) revealed white blood cell (WBC) count 12×10^9^/L, lymphocyte%: 78.34%, hemoglobin (Hb) 107g/L and platelets 100×10^9^/L, lactate dehydrogenase(LDH) level was 627 U/L, virologic profile demonstrated HbsAg(-), HbsAb(+), HBeAb (+), HBcAb (+) and EBV DNA < 5000/ml. A contrast-enhanced computed tomography (CT) scan of the chest and abdomen showed obvious splenomegaly without lymphadenopathy. Bone marrow aspirate reflected increasing mature lymphocytes suggesting lymph proliferative disorders. During local admission, the patient received symptomatic treatment including antibiotics and glucocorticoid, but no response was observed. The patient subsequently was transferred to our hospital for further diagnosis and treatment. Physical examination showed massive splenomegaly without general enlargement of the lymph nodes, ECOG was 4 points. The CBC revealed WBC: 16.61×10^9^/L, neutrophil: 0.93×10^9^/L, Hb: 70g/L, PLT: 55×109/L, ferritin level was 608.70ng/mL and soluble CD25 was 7245 IU/ml. Bone marrow aspirate and biopsy showed lymphocytosis of LGL cells which represented medium to large cells with eccentric nuclei and abundant cytoplasm containing course azurophilic granules, accounting for 23% (Figure 1). Immunohistochemistry analysis of bone marrow showed malignant cells that were CD3 (+), CD56 (-), TIA1 (+), granzyme B (-). Flow cytometry illustrated abnormal T-cell immunophenotype was CD3+CD4-CD8- CD5-CD7-, TCRαβ+ and further 24 subtypes of the variable region families of β chain was less than 30% which is an indirect proof of T cell clonality abnormality. TCR gene rearrangement by PCR was positive for TCR β and γ (Figure 2), STAT3 mutation identified by Sanger sequencing was negative. Chromosomal alterations detected by conventional cytogenetics showed 49, XY, +5, +13, +14, -16, der (16), +22 [4cp]/46, XY [6]. And the patient, therefore, with typical cellular morphology, monoclonal immunophenotype and TCR gene rearrangement, a diagnosis of T-LGL leukemia was established. Besides T-LGL leukemia, the patient, with prolonged fever, pancytopenia, splenomegaly, hyperferritinemia and increasing level of sCD25 which also reached diagnostic criterion of HLH-2004 protocol of hemophagocytic lymphohistiocytosis. Considering the patient, with awful physical condition, pursued an aggressive clinical course with HLH, intensive regimen CEOP (cyclophosphamide with a dose of 750mg/m^2^ d1, vincristine 2mg d1, etoposide 100mg/m^2^ d1-d3, prednisone 60mg/m^2^×d for five days) instead of oral MTX plus prednisone was recommended. After two cycles of chemotherapy, the patient was discharged from the hospital without fever and abdominal pain. But he showed a recurrence of systemic symptoms and intermittent dizziness two weeks after discharge, then he readmitted to our hospital. CT scan of brain indicated no space-occupying lesions, but cerebrospinal fluid test showed that cell count was 540 /μl, Pandy test was positive, protein in cerebrospinal fluid was 0.95g/L, hypercellular fluid was infiltrated with mononuclear cells which presented with abundant cytoplasm and azurophilic granules (Figure 3), with its immunophenotype in accordance with bone marrow, the diagnosis of T-LGL leukemia with CNS involvement was confirmed. The patient relieved of dizziness after intrathecal injection of methotrexate, cytarabine and dexamethasone was administrated but recurrence of dizziness occurred only three days later. Given that the disease progressed with CNS involvement during chemotherapy and the patient with fragile physical condition, intensive intravenous chemotherapy was not appropriate. Into this embarrassing situation, oral metronomic regimen T-PEPC (oral administration of prednisone 40mg, cyclophosphamide 50mg, etoposide 50mg, methyhydrazine 50mg, thalidomide 100mg every day) targeted both malignant cells and microenvironment was considered to control progressive disease with low toxicities. About one month after receiving T-PEPC regimen, body temperature of the patient returned to normal range and a significant improvement in abdominal pain was achieved. The latest follow-up CBC test revealed WBC: 3.5×10^9^/L, lymphocyte%: 38%, Hb: 109g/L, PLT: 290×10^9^/L without CNS symptoms and normal size of spleen, and the patient remained symptom-free at 8-month follow-up. This study was approved by the ethics committee of the the First Affiliated Hospital of Nanjing Medical University.

## DISCUSSION

Granular lymphocytes are normally accounted for 10%-15% of peripheral blood mononuclear cells with abundant cytoplasms and azurophilic granules [6]. The diagnosis of LGL leukemia is based on chronic LGL peripheral blood expansion ( > 2×10^9^/L), usually lasting for more than 6 months with typical morphological characteristics. Semenzato et al [7] studied 11 patients with chronic granular lymphocytosis that did not meet the criteria for LGL leukemia, namely, all patients had a LGL peripheral blood expansion less than 2×10^9^/L, however, the clinical and laboratory features of these patients are similar to those patients with LGL cells greater than 2×10^9^/L. Besides, immunphenotype, TCR gene rearrangement and follow-up clinical course of these patients all meet the diagnosis of LGL leukemia. Thus, current diagnosis of LGL leukemia could be refined as 1) chronic LGL peripheral blood expansion (mostly 2-20×10^9^/L), usually lasting for more than 6 months, while 25%-30% patients with circulating LGL cells less than 0.5×10^9^/L need assistant diagnosis with immunophenotype and TCR gene rearrangement. 2) Classical immunophenotype mostly CD3^+^CD8^+^CD57^+^TCRαβCD3+CD4+CD8-CD57+ TCRαβ+, CD3+CD4-CD8-CD57+TCRγδ+. 3) T-cell clonality demonstrated by rearrangement of TCRγ gene using PCR or vβ-expression *via* flow cytometry [7–8]. 4) Clinical presentation like cytopenia, splenomegaly and rheumatoid arthritis etc. More importantly, diseases like HIV infections, post-splenectomy, allogeneic bone marrow transplantation, or solid organ transplantation could present with benign LGL cells proliferations with similar morphology [9–12]. After the widespread availability of TCR and flow cytometry, it became much accurate to distinguish the LGL leukemia as a neoplastic disease or reactive lymphocytosis [13]. According to current criteria, the patient we reported, with typical morphological feature, unique immunophenotyping by flow cytometry and T cell clonality demonstrated by TCR rearrangement plus clinical presentation, could make a definite diagnosis of T-LGL leukemia.

Recently, Hanna L.M. et. al [14] reported mutations in the signal transducer and activator of transcription 3 gene (STAT3) were found in 31 of 77 patients (40%) with LGL leukemia, recurrent mutational hot spots included Y640F, D661V, D661Y,N647. Among them, the most frequent mutation hot spots were Y640F and D661V. Besides, STAT3 mutation was also found in CLPD-NK cohorts. Comparison of clinical characteristics of these patients showed that neutropenia and rheumatoid arthritis were more common among patients with a STAT3 mutation. Besides, patients with STAT3 mutations also tended to need more treatments like corticosteroids, cyclosporine, cyclophosphamide or red blood cell transfusion [15], suggesting that aberrant STAT3 signaling underlies the pathogenesis of this disease. Thus STAT3 can be served as an auxiliary diagnosis tool of classifying LGL leukemia as true T-cell leukemia or hyperreactive or persistent T-cell response. STAT3 was only found in the T-cell (CD8+) fraction, and expression analysis demonstrated that patients with or without STAT3 mutations had many common overexpressed STAT3 genes [16]. STAT3 mutation status of this patient is negative, which may be related to atypical immunophenotyiping of CD3+CD4-CD8-.

To our knowledge, this is the first case that reported a patient of T-LGL leukemia with phenotype of TCRαβ﹢CD3+CD4-CD8- presented with an aggressive clinical course and CNS involvement during intensive treatment. A vast majority of patients (80%-90%) with T-LGL leukemia showed a CD3+ CD4-CD8+ CD57+ CD56- CD28-,TCRαβphenotype [17], while CD4- CD8- usually accompanied with TCR γδ, but had a favorable survival of 85% at 3 years [18]. To our knowledge, T-LGL leukemia is a rare lymphoproliferative disorder of mature cytotoxic CD3+ lymphocytes with a clinical indolent course and a median survival of 10 years [8, 19, 20]. Aggressive clinical course usually exists in NK-LGL leukemia, another type of LGL leukemia, while NK cells do not express CD3 or TCR, whose TCR genes are not rearranged [21]. Of course, T-LGL leukemia can present with a severe systemic illness that is rapidly progressive and resistant to treatment but with a phenotype of CD3+ and CD56+, which is separate from the usual CD3+, CD56- T-LGL leukemia [22] Aggressive T-cell LGL leukemia is supposed to arise from clonal evolution of indolent T-cell LGL leukemia but it is more like a de novo development [23, 24].The patient in our case is also the typical CD3+CD56-, with a unique phenotype variants of dual CD4-/CD8-,while it commonly accompanied with γδ+ T-cells [25]. Therefore, whether TCRɑβ with phenotype variants of dual CD4-/CD8- could lead to an aggressive clinical course and resistant to intravenous chemotherapy warrants further investigation, besides, we should exclude other aggressive LGL leukemia, that's why we studied STAT5b status in this patient, and the result is negative. As we know, STAT5b is a vital regulator in the proliferation, differentiation and survival of tumor cell and other aggressive lymphoid maligancies harboured STAT5b mutations. The result, which to some extent, made our case more complicated.

The clinical presentation of indolent T-LGL leukemia embodies about one third as asymptomatic and two thirds symptomatic with neutropenia resulting in an increased frequency of bacterial infection, anemia, thrombocytopenia, splenomegaly and autoimmune conditions like rheumatoid arthritis, pure red cell aplasia, immune thrombocytopenia purpura, and the prognosis of these individuals is usually favorable with immunosuppressive regimen [26]. Aggressive T-LGL leukemia is relatively rare with clinical features including cytopenia, acute B symptoms such as fever, night sweats, weight loss and hepatosplenomegaly [27, 28]. The patient we reported herein pursued an aggressive clinical course with high fever, splenomegaly, and increasing process with CNS involvement during intravenous chemotherapy but without any indications of autoimmune conditions.

Since the etiology of T-LGL leukemia is not clear and no prospective clinical trials have been reported, thus, treatment recommendations are based on case reports and retrospective studies [6]. Most patients are treated with low dose immunosuppressive therapy regarding the indolent clinical course, which include single agents like methotrexate, oral cyclophosphamide or cyclosporine and often combine with prednisone, and the treatment only started in those patients with severe neutropenia, anemia or rheumatoid arthritis (RA) [8]. Since prednisone can temporarily improve neutropenia, MTX has been an effective treatment for RA and cyclophosphamide has been preferentially used in LGL leukemia patients with pure red cell aplasia [29, 30]. Besides, such immunosuppressive agents, like purine analogue (e.g. fludarabine in combination with dexamethasone) have had impressive response rate and Alemtuzumab (Campath), as a monoclonal antibody against CD52 is served as an effective therapy for LGL leukemia mostly with PRCA [31]. Furthermore, recent findings suggested that patients harboring the Y640F mutation in STAT3 respond better to methotrexate. For this patient we report herein, with aggressive clinical presentation, intravenous injection of CHOP-like chemotherapy may be a better option [32], but unfortunately, intravenous CEOP chemotherapy didn't achieve a satisfactory outcome, and disease progression with CNS involvement occurred during treatment. Considering the patient who has got aggressive disease with poor physical condition, could not sustain further intravenous chemotherapy, metronomic regimen T-PEPC, as an alternative was administrated.

The term metronomic chemotherapy was originally coined in an editorial published in 2000 by Hanahan et al. [33], referring to the frequent, even daily administration of cytotoxic drugs at comparatively low doses with minimal or prolonged drug-free breaks [34]. As it can maintain modest and acceptable toxicity profiles plus lower cost resulting from fewer side-effect associated expenditures and the usage of inexpensive oral drugs such as cyclophosphamide compared with maximum tolerated dose (MTD) regimens, have gained an increasing popularity nowadays [35]. Furthermore, methyhydrazine and thalidomide can infiltrate through the blood brain barrier, which can control potential CNS infiltration. This patient remained symptom-free and hematologic remission in the latest follow-up for more than 8 months, which suggests a better control of both systemic and CNS diseases. Taken together, this is the first case of aggressive T-LGL leukemia with phenotype of TCRαβ and CD4-/CD8- which shows a CNS involvement during chemotherapy and responded well to T-PEPC metronomic regimen, suggesting that metronomic regimen should be a better treatment option for such patients.

## CONCLUSIONS

T-LGL leukemia usually is an indolent disease. Here we reported a patient of aggressive T-LGL leukemia accompanied with a unique immunophenotype of CD3+CD4-CD8- CD5-CD7-TCRαβ+, and clinical manifestations were lasting fever, splenomegaly and HLH symptom. The patient therefore started a chemotherapy of CEOP but pursued a disease progression and CNS involvement during treatment. Thus T-PEPC metronomic regimen was administrated as an alternative, considering the elderly patient with fragile physical condition. The patient gained a satisfactory outcome with such treatment and remained 8-month symptom-free. Therefore, from this case, we can conclude that T-LGL leukemia patients with unique TCRɑβ, and phenotype variants of dual CD4-/CD8- may pursue an aggressive clinical course and for elderly patient that cannot sustain an intensive intravenous chemotherapy, a relatively moderate chemotherapy like metronomic regimen should be recommended.

## CONSENT

Written informed consent was obtained from the patient's family for publication of this case report and any accompanying images. A copy of the written consent is available for review by the Editor-in-Chief of this journal.

## Abbreviations

HLH: hemophagocytic lymphohistiocytosis
CNS: central nervous system
LGLL: Large granular lymphocytic leukemia
RA: rheumatoid arthritis
CBC: Complete blood cell
WBC: white blood cell
CT: contrast-enhanced computed tomography.
